# Sex differences in the association of the CRP-TyG index with coronary artery disease and long-term mortality in MASLD

**DOI:** 10.3389/fendo.2026.1864536

**Published:** 2026-07-28

**Authors:** Lin Shi, Sen Wang, Juan Du, Renwei Guo

**Affiliations:** 1 Department of Gastroenterology, Xuzhou Central Hospital, Southeast University, Xuzhou, Jiangsu, China; 2 Department of Cardiology, Xuzhou Central Hospital, Southeast University, Xuzhou, Jiangsu, China; 3 Department of Neurology, Xuzhou Central Hospital, Southeast University, Xuzhou, Jiangsu, China

**Keywords:** coronary artery disease, CRP-triglyceride glucose index, metabolic dysfunction-associated steatotic liver disease, prognosis, risk stratification, sex differences

## Abstract

**Background:**

Metabolic dysfunction-associated steatotic liver disease (MASLD) increases cardiovascular risk, but non-invasive tools for risk stratification are limited. The C-reactive protein–triglyceride–glucose (CTI) index integrates metabolic and inflammatory pathways, but its value in MASLD is unclear.

**Methods:**

We examined the association between CTI and obstructive coronary artery disease (CAD) in MASLD patients who underwent coronary computed tomography angiography. Machine-learning-assisted feature selection was used to assess the relative stability of CTI among candidate clinical variables, and multivariable logistic regression was used to evaluate its independent association with obstructive CAD. Discriminative performance was assessed using ROC curves, calibration analysis, clinical impact analysis, and restricted cubic spline analysis, with sex-stratified and sensitivity analyses. The prognostic relevance of CTI for all-cause and cardiovascular mortality was further evaluated in a population-based NHANES MASLD cohort.

**Results:**

Higher CTI quartiles correlated with greater metabolic burden and CAD prevalence (P < 0.001). CTI was independently associated with obstructive CAD (OR = 1.68, 95% CI 1.33–2.12), with a graded association across quartiles. CTI showed moderate apparent discrimination for obstructive CAD, with higher AUCs than FIB-4, TyG-BMI, and TyG in the overall, male, and female populations. In the NHANES cohort, elevated CTI was associated with increased risks of all-cause and cardiovascular mortality, with the association for cardiovascular mortality being more evident in men than in women after full adjustment.

**Conclusion:**

CTI was independently associated with CCTA-defined obstructive CAD in a high-risk MASLD cohort and showed prognostic relevance for long-term mortality in a population-based MASLD cohort.

## 1 Introduction

Metabolic dysfunction–associated steatotic liver disease (MASLD), recently redefined from nonalcoholic fatty liver disease, is characterized by hepatic steatosis in conjunction with at least one cardiometabolic risk factor (1). It has become the most prevalent chronic liver disease worldwide, paralleling the global epidemics of obesity and type 2 diabetes (2). Beyond hepatic outcomes, MASLD substantially increases cardiovascular risk, and cardiovascular disease (CVD) has emerged as the leading cause of mortality in this population (3–6). This excess risk is driven by shared pathophysiological mechanisms, including insulin resistance, chronic inflammation, and atherogenic dyslipidemia (7, 8). Despite this interconnection, cardiovascular risk in MASLD remains frequently underestimated, underscoring the need for integrated heart–liver risk assessment (6, 9).

Coronary computed tomography angiography (CCTA) enables detailed assessment of coronary artery disease (CAD) but is limited by cost, radiation exposure, and availability, making it unsuitable for widespread screening (10–12). Non-invasive fibrosis scores, such as FIB-4, are widely used for hepatic risk stratification and have shown some association with cardiovascular outcomes (6, 13). However, these indices primarily capture fibrotic burden and incompletely reflect the systemic metabolic-inflammatory disturbances that drive atherosclerosis in MASLD.

The CTI is a composite biomarker that captures metabolic-inflammatory dysregulation by integrating a surrogate of insulin resistance (TyG) with a marker of systemic inflammation (CRP) (14–17). While CTI has been associated with cardiovascular events in general populations (14–17), its specific diagnostic value for CCTA-confirmed obstructive CAD in patients with MASLD remains unexplored.

Furthermore, cardiovascular risk in MASLD is modulated by sex differences in metabolism and inflammation, which may influence biomarker performance (18–20). Therefore, this study aimed to: (1) evaluate the independent association and discriminative ability of CTI for obstructive CAD in a CCTA-defined MASLD cohort, with particular attention to potential sex-related differences; and (2) evaluate the prognostic relevance of CTI for long-term all-cause and cardiovascular mortality in a population-based MASLD cohort.

## 2 Methods

### 2.1 Study design and data sources

This study was conducted in two phases. The first phase evaluated the association between CTI and obstructive CAD in a CCTA-defined MASLD cohort from Xuzhou Central Hospital (January 2020–December 2023). The second phase evaluated the prognostic relevance of CTI for mortality in a population-based MASLD cohort from the National Health and Nutrition Examination Survey (NHANES) 2007–2010.

### 2.2 Participants – imaging-based MASLD cohort

Consecutive adult patients who underwent CCTA during this period were screened for eligibility at Xuzhou Central Hospital between January 2020 and December 2023 (10, 21). The study protocol adhered to the Declaration of Helsinki and was approved by the institutional ethics committee of Xuzhou central hospital (Approval No. XZXY-LK-20251222-0140). Written informed consent was waived due to the retrospective design and use of deidentified data.

Exclusion criteria included: (1) a history of significant alcohol consumption or other chronic liver diseases (e.g., viral hepatitis, autoimmune hepatitis, hemochromatosis, Wilson’s disease); (2) a known history of coronary artery disease, prior myocardial infarction (PMI), coronary revascularization, or heart failure (HF); (3) incomplete clinical, biochemical, or imaging data; and (4) poor CCTA image quality precluding reliable assessment of coronary stenosis.

A total of 8,571 patients who underwent CCTA for suspected coronary artery disease were initially screened. After exclusions, 2,119 MASLD patients were included (**Supplementary Figure 1**).

### 2.3 Participants – external prognostic validation

Data for external prognostic validation were obtained from NHANES 2007–2010 (21, 22). Adults aged ≥20 years with complete data for CTI calculation and meeting MASLD criteria (excluding excessive alcohol intake or other liver diseases) were included, yielding 1,187 participants. All NHANES protocols were approved by the NCHS Ethics Review Board, with written informed consent obtained from all participants (21, 22).

### 2.4 Clinical and biochemical data collection

Demographic information included age and sex. Lifestyle behaviors were recorded through structured interviews and encompassed smoking and alcohol consumption status. Clinical history was obtained from medical records, covering physician-diagnosed hypertension, diabetes mellitus, and hyperlipidemia. Anthropometric indices such as body mass index (BMI), systolic blood pressure (SBP), and diastolic blood pressure (DBP) were measured by trained nurses using standardized protocols.

Venous blood samples were drawn in the morning after an overnight fast of at least 8 hours. The following parameters were determined: white blood cell count (WBC), platelet count (PLT), neutrophil count (NEUT), lymphocyte count (LYM), fasting plasma glucose (FPG), glycated hemoglobin (HbA1c), total cholesterol (TC), triglyceride (TG), low-density and high-density lipoprotein cholesterol (LDL-C, HDL-C), alanine aminotransferase (ALT), aspartate aminotransferase (AST), albumin (ALB), total bilirubin (TBIL), γ-glutamyl transferase (GGT), total bile acid (TBA), uric acid (UA), creatinine (Cr), and C-reactive protein (CRP).

Several composite indices were calculated to reflect metabolic or hepatic function, including the triglyceride-glucose index (TyG), TyG-BMI, fibrosis-4 index (FIB-4), and the CRP-TyG index (CTI). The exposure-related indices in this study were calculated as follows: CTI = 0.412 × Ln (CRP [mg/L]) + Ln (TG [mg/dL] × FPG [mg/dL]/2); TyG = Ln (TG [mg/dL] × FPG [mg/dL]/2); TyG–BMI = TyG × BMI; FIB-4 = (Age [years] × AST [U/L]) ÷ (PLT [10^9^/L] × √ALT [U/L]). All laboratory measurements were obtained after an overnight fast. When TG or FPG were recorded in mmol/L, they were converted to mg/dL using TG (mg/dL) = TG (mmol/L) × 88.57 and FPG (mg/dL) = FPG (mmol/L) × 18.02 before calculation. All measurements followed uniform quality control procedures to ensure reproducibility and minimize analytical bias.

### 2.5 CCTA assessment and coronary stenosis

CCTA assessment was performed exclusively in the imaging-based MASLD cohort. All procedures for CCTA and coronary artery calcium scanning were performed in accordance with the guidelines of the Society of Cardiovascular Computed Tomography on a dedicated dual-source CT scanner (SOMATOM Definition, Siemens Healthineers, Germany) (10). Detailed CT scanning protocols are provided in the **Supplementary Materials**.

All CT images were independently evaluated by two experienced radiologists who were blinded to the clinical data of the patients. Discrepant cases were adjudicated by a third senior radiologist. CAD severity was assessed based on these evaluations. The primary outcome of this study was obstructive CAD, defined as the presence of ≥50% diameter stenosis in any of the four major epicardial coronary arteries as detected by CCTA.

### 2.6 Diagnosis of MASLD

Overall, liver ultrasonography was the primary imaging modality for diagnosing hepatic steatosis, accounting for 92.8% of cases, while CT-based assessment was used for the remaining 7.2% (1, 2). Ultrasonographic hepatic steatosis was diagnosed by experienced sonographers based on standard imaging features, including increased hepatic echogenicity, hepatorenal echo contrast, vascular blurring, and posterior beam attenuation. For participants without available liver ultrasonography, clinically available CT images were reviewed as supplementary imaging evidence. These included abdominal CT examinations when available, or liver-visible CT images obtained during the CCTA examination when the hepatic parenchyma was sufficiently covered for assessment. MASLD was defined as imaging-confirmed hepatic steatosis with at least one cardiometabolic risk factor, after excluding excessive alcohol intake and other known causes of chronic liver disease, including viral hepatitis, autoimmune liver disease, and other specific liver diseases (1, 2). To assess inter-rater reliability, a random subset of imaging examinations was independently re-evaluated by two experienced readers who were blinded to laboratory biomarkers, CTI values, and CAD status. Agreement for the presence of hepatic steatosis was assessed using Cohen’s kappa coefficient. Detailed diagnostic criteria can be found in the **Supplementary Materials**.

In the NHANES cohort, hepatic steatosis was defined using the United States Fatty Liver Index (US-FLI), a validated non-invasive score developed from NHANES data and calibrated against ultrasound-detected hepatic steatosis (23–25). Participants with a US-FLI value ≥30 were considered to have hepatic steatosis. MASLD was then defined as US-FLI-defined hepatic steatosis in the presence of at least one cardiometabolic risk factor, after excluding participants with excessive alcohol consumption, viral hepatitis, or other identifiable causes of chronic liver disease. The calculation of US-FLI and its diagnostic performance have been described in previous studies (23).

### 2.7 Feature selection and stability analysis

To identify robust candidate features associated with obstructive CAD, we applied four complementary feature selection algorithms: LASSO regression (glmnet package, 10-fold cross-validation), Boruta (500 iterations), XGBoost (binary logistic objective), and SVM-RFE (linear kernel) (26–28). These algorithms were used for feature selection and stability assessment rather than for the development of a standalone predictive model. To evaluate stability, we performed 1,000 subsampling iterations; in each iteration, 70% of the data were randomly selected, and all four algorithms were executed. Selection frequency across resamples was calculated for each variable, and variables with high aggregated selection frequencies were considered stable. Detailed algorithm parameters are provided in the **Supplementary Methods**.

### 2.8 Mortality in NHANES

Mortality status in NHANES was determined through linkage to the National Death Index through December 31, 2019. All-cause mortality was defined as death from any cause; CVD mortality was identified using ICD codes corresponding to cardiovascular causes of death.

### 2.9 Statistical analysis

Statistical analyses for cross-sectional outcomes were performed in the imaging-based cohort, while time-to-event analyses were conducted in the NHANES cohort. In imaging-based cohort, patients were divided into four groups according to the quartiles of CTI (Q1: < 9.61; Q2: 9.61–9.97; Q3: 9.97–10.35; Q4: > 10.35). In NHANES cohort, individuals were divided into survivors and non-survivors. Descriptive statistics were presented according to data type and distribution. Continuous variables were summarized as mean ± standard deviation or median (interquartile range), and compared using one-way analysis of variance (ANOVA) or the Kruskal–Wallis test, as appropriate. Categorical variables were expressed as frequencies (percentages) and compared using Pearson’s chi-squared test or Fisher’s exact test. For the NHANES cohort, all analyses accounted for the complex survey design using sampling weights, strata, and primary sampling units per National Center for Health Statistics guidelines.

Candidate features identified through multiple feature selection algorithms were used to inform covariate adjustment in multivariable logistic regression models. Multicollinearity was assessed using variance inflation factors (VIF); variables with VIF > 10 were considered collinear and handled by exclusion. Variables with high selection frequency (top 20 variables), together with clinically relevant covariates, were incorporated into three progressively adjusted models (Model 1–3). Model 1 was unadjusted; Model 2 adjusted for age and sex; and Model 3 further adjusted for LDL-C, diabetes, SBP, HDL-C, NEUT, FIB-4, WBC, LYM, DBP, AST, ALT, and hypertension.

To assess the ability of the CTI index to discriminate patients with CAD, receiver operating characteristic (ROC) curves and the corresponding areas under the curve (AUCs) with 95% confidence intervals were calculated and compared using DeLong’s test. Model calibration was evaluated through calibration plots comparing predicted and observed probabilities. Restricted cubic spline (RCS) analysis was performed to explore potential nonlinear relationships between CTI and CAD risk.

Clinical utility was further evaluated using clinical impact curves (CIC). The incremental discriminative ability of CTI beyond other indices was further examined using the net reclassification improvement (NRI) and integrated discrimination improvement (IDI) indices. To explore potential sex-related differences, ROC, NRI, and IDI analyses were additionally conducted in male and female subgroups.

Sensitivity analyses were performed to assess the robustness of the main association by redefining obstructive CAD as ≥70% luminal stenosis. Subgroup analyses were additionally conducted according to age, diabetes status, hypertension status, and sex, using the same covariate-adjustment strategy as the primary model when applicable.

For the prognostic analysis in the NHANES cohort, survival time was calculated from the date of NHANES examination to the date of death or the end of follow-up, whichever occurred first. Cox proportional hazards regression models were constructed to estimate HRs and 95% CIs for all-cause mortality and CVD mortality according to CTI. The proportional hazards assumption was assessed using Schoenfeld residuals.

All statistical analyses were performed using R software (version 4.4.0). A two-sided P value < 0.05 was considered statistically significant.

## 3 Results

### 3.1 Baseline characteristics of the study population

In the randomly selected subset, inter-rater agreement for the diagnosis of hepatic steatosis was high, with a Cohen’s kappa coefficient of 0.83. **Table 1** summarizes the baseline characteristics of 2,119 MASLD patients grouped by CTI quartiles. The mean age was 52.7 years, and 56.9% were male. Higher CTI quartiles were associated with a less favorable cardiometabolic profile, including higher BMI, blood pressure, glucose, triglycerides, TyG, and inflammatory markers (WBC, NEUT, and CRP), and lower HDL-C. The prevalence of hypertension, diabetes, and hyperlipidemia increased progressively across quartiles, as did the rate of obstructive CAD.

**Table 1:** Baseline characteristics according to CTI quartiles.

Variables	Total (n = 2119)	Q1 (n = 529)	Q2 (n = 530)	Q3 (n = 530)	Q4 (n = 530)	P-value
Age, years	52.69 ± 14.41	50.90 ± 13.40	52.92 ± 16.15	53.07 ± 13.37	53.85 ± 14.39	0.007
Sex, n(%)						<0.001
Female	913 (43.09)	246 (46.50)	248 (46.79)	235 (44.34)	184 (34.72)	
Male	1206 (56.91)	283 (53.50)	282 (53.21)	295 (55.66)	346 (65.28)	
BMI	25.57 ± 1.81	24.25 ± 1.85	25.42 ± 1.65	25.98 ± 1.45	26.63 ± 1.35	< 0.0001
SBP, mmHg	132.37 ± 0.51	128.02 ± 1.20	132.23 ± 1.64	133.33 ± 2.20	133.78 ± 1.23	0.01
DBP, mmHg	67.81 ± 0.37	65.42 ± 0.97	65.86 ± 0.76	68.82 ± 1.39	68.72 ± 0.85	0.005
Smoking, n(%)	786 (37.1)	160 (30.2)	207 (39.1)	223 (42.1)	196 (37.0)	<0.001
Medical history, n(%)						
Hypertension	1444 (68.1)	239 (45.2)	364 (68.7)	406 (76.6)	435 (82.1)	< 0.0001
Diabetes	836 (39.4)	81 (15.3)	172 (32.5)	259 (48.9)	324 (61.1)	< 0.0001
Hyperlipidemia	473 (22.3)	59 (11.2)	103 (19.4)	131 (24.7)	180 (34.0)	< 0.0001
Laboratory test						
WBC(× 109/L)	7.55 ± 0.05	6.59 ± 0.19	7.22 ± 0.14	7.61 ± 0.19	7.88 ± 0.13	< 0.0001
PLT(× 109/L)	228.01 ± 1.63	209.94 ± 4.02	222.92 ± 4.74	226.28 ± 3.94	237.07 ± 4.43	< 0.0001
NEUT(× 109/L)	4.59 ± 0.04	3.91 ± 0.09	4.42 ± 0.11	4.56 ± 0.10	4.89 ± 0.10	< 0.0001
LYM(× 109/L)	2.05 ± 0.03	1.84 ± 0.14	1.91 ± 0.09	2.13 ± 0.12	2.05 ± 0.05	0.09
FPG, mmol/L	6.80 ± 3.09	5.25 ± 1.01	5.79 ± 1.52	6.68 ± 2.32	9.50 ± 4.33	<0.001
HbA1c, %	7.48 ± 1.89	6.27 ± 0.98	6.85 ± 1.40	7.35 ± 1.62	8.46 ± 2.10	<0.001
TC, mmol/L	4.86 ± 1.26	4.40 ± 1.02	4.75 ± 1.03	4.88 ± 1.07	5.42 ± 1.60	<0.001
TG, mmol/L	1.85 ± 1.27	1.05 ± 0.84	1.65 ± 1.39	2.36 ± 1.88	3.80 ± 2.75	<0.001
LDL, mmol/L	2.91 ± 0.87	2.69 ± 0.82	2.96 ± 0.82	2.98 ± 0.85	3.03 ± 0.96	<0.001
HDL, mmol/L	1.04 ± 0.30	1.11 ± 0.29	1.07 ± 0.28	1.04 ± 0.27	0.95 ± 0.32	<0.001
ALT, U/L	31.00 (20.80, 48.00)	28.00 (19.00,44.00)	30.00 (20.00,46.52)	32.10 (22.00,50.92)	32.00 (21.10,51.60)	<0.001
AST, U/L	23.30 (18.00, 32.77)	23.00 (18.00,30.90)	23.00 (18.00,32.00)	24.00 (19.00,33.00)	24.00 (18.20,34.00)	0.003
ALB, g/L	42.68 ± 4.64	42.09 ± 4.81	42.80 ± 4.50	42.97 ± 4.45	42.86 ± 4.74	<0.001
TBIL, umol/L	13.19 ± 14.42	13.88 ± 14.10	13.33 ± 16.86	12.78 ± 12.06	12.76 ± 14.23	0.140
GGT, U/L	37.95 (25.00, 61.95)	29.90 (21.00,48.10)	34.00 (24.00,57.00)	40.00 (28.00,61.00)	48.00 (32.00,80.00)	<0.001
TBA, umol/L	3.50 (2.00, 5.80)	3.10 (1.90,5.40)	3.40 (1.90,5.80)	3.60 (2.10,5.80)	3.70 (2.10,6.10)	0.001
UA, umol/L	345.00 (280.00, 414.00)	327.00 (264.75,395.00)	339.90 (279.10,405.00)	356.00 (291.00,428.00)	357.00 (285.00,428.00)	<0.001
Creatinine, umol/L	64.00 ± 35.56	64.39 ± 45.25	64.08 ± 27.38	63.31 ± 20.48	64.21 ± 42.95	0.872
CRP, mg/L	6.22 ± 0.03	5.73 ± 0.04	5.97 ± 0.06	6.14 ± 0.08	7.18 ± 0.13	< 0.0001
TyG	8.86 ± 0.03	8.04 ± 0.02	8.59 ± 0.01	9.02 ± 0.01	9.78 ± 0.03	< 0.0001
TyG-BMI	226.55 ± 16.3	194.97 ± 14.9	218.36 ± 14.2	234.34 ± 13.1	260.44 ± 12.2	< 0.0001
FIB-4	1.34 ± 0.82	0.89 ± 0.41	1.06 ± 0.52	1.14 ± 0.61	1.66 ± 1.15	< 0.0001
CAD, n(%)	495 (23.3)	71 (13.4)	101 (19.1)	137 (25.8)	186 (35.1)	< 0.0001

Sex-stratified characteristics (**Supplementary Table 1**) showed that men had higher CTI, triglycerides, LDL-C, and CAD prevalence, whereas women had higher HDL-C and platelet counts, indicating a greater cardiometabolic burden in men.

### 3.2 Feature selection and variable stability analysis

**Figure 1:**
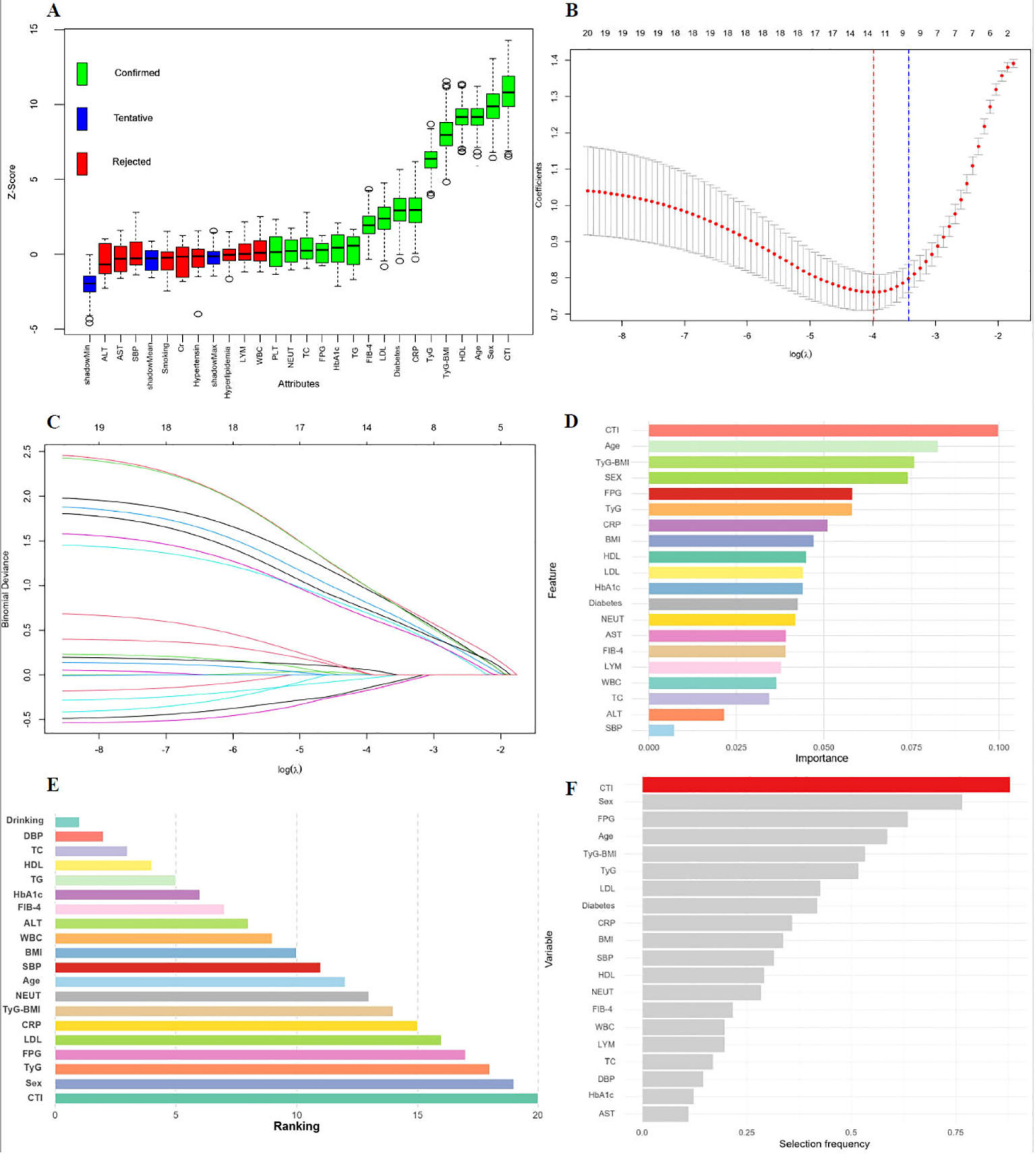
Feature selection results using four complementary algorithms and frequency-based stability analysis. **(A)** Boruta algorithm: Boxplots showing feature importance based on Z-scores. Variables classified as confirmed (green) were deemed significant contributors, tentative (blue) variables were borderline, and rejected (red) variables showed minimal relevance to obstructive CAD. **(B–C)** LASSO regression: **(B)** Ten-fold cross-validation identifying the optimal regularization parameter (λ) minimizing binomial deviance; **(C)** LASSO coefficient profiles of included variables. **(D)** XGBoost model: Feature importance ranking generated by the XGBoost algorithm. The 20 most influential predictors are displayed in descending order of importance, with lower ranks indicating higher relative contribution. **(E)** SVM-RFE algorithm: Top 20 retained variables identified by the SVM–recursive feature elimination method. Variables with higher ranking values were eliminated later in the iterative selection process, indicating greater relevance. **(F)** Summary of the top 20 predictors based on aggregated feature selection frequencies across 1,000 bootstrap iterations, integrating results from all four algorithms.

Feature selection analyses were performed to evaluate the relative stability of candidate variables associated with obstructive CAD rather than to construct a standalone prediction model. Four complementary algorithms—LASSO, Boruta, XGBoost, and SVM-RFE—were used to capture different aspects of feature relevance. Across four complementary feature selection algorithms (LASSO, Boruta, XGBoost, and SVM-RFE), CTI consistently ranked among the most influential features for obstructive CAD (**Figures 1A–E**). In 1,000 subsampling iterations, CTI demonstrated the highest aggregated selection frequency, exceeding 75% (**Figure 1F**), supporting its stability across feature selection procedures. Sex-stratified analyses revealed that CTI was consistently selected in both men and women, with selection frequencies exceeding 70% (**Supplementary Figures 2A, B**). However, the accompanying top-ranked features differed by sex, CTI, TG, age, FPG, and CRP predominated, reflecting a metabolic-inflammatory profile; in women, age, CTI, LDL-C, TyG, and BMI were most prominent, indicating an age–lipid–adiposity pattern (**Supplementary Figures 2C, D**).

### 3.3 Association between CTI and obstructive CAD in overall and sex-stratified populations

Multivariable logistic regression showed a significant association between CTI and obstructive CAD (**Table 2**). Per one-unit increase in CTI, the fully adjusted odds of CAD were elevated by 68% (OR 1.68, 95% CI 1.33–2.12, P < 0.001), with a graded association across quartiles (Q4 vs. Q1: OR 2.66, 95% CI 2.03–3.42; P for trend <0.001).

**Table 2:** Multivariate-adjusted odd ratios (95% confidence intervals) of CTI for obstructive coronary artery disease in MASLD.

Variables	ORs (95%CI), P-value		
	Model 1	Model 2	Model 3
Overall			
CTI as continuous	1.82 (1.45-2.28), <0.0001	1.74 (1.38-2.19), <0.0001	1.68 (1.33-2.12), <0.0001
CTI quartile			
Q1	1.00	1.00	1.00
Q2	1.42 (1.08-1.87), 0.012	1.35 (1.02-1.78), 0.035	1.28 (0.97-1.69), 0.083
Q3	2.15 (1.66-2.78), <0.0001	1.92 (1.48-2.49), <0.0001	1.76 (1.35-2.29), <0.0001
Q4	3.48 (2.71-4.47), <0.0001	2.94 (2.28-3.79), <0.0001	2.66 (2.03-3.42), <0.0001
P for trend	<0.0001	<0.0001	<0.0001
Male			
CTI as continuous	2.04 (1.58-2.63), <0.0001	1.89 (1.46-2.45), <0.0001	1.82 (1.40-2.36), <0.0001
CTI quartile			
Q1	1.00	1.00	1.00
Q2	1.58 (1.15-2.17), 0.005	1.46 (1.06-2.01), 0.020	1.38 (1.00-1.91), 0.051
Q3	2.48 (1.83-3.36), <0.0001	2.14 (1.57-2.91), <0.0001	1.96 (1.43-2.76), <0.0001
Q4	4.12 (3.08-5.51), <0.0001	3.42 (2.55-4.59), <0.0001	3.28 (2.21-4.12), <0.0001
P for trend	<0.0001	<0.0001	<0.0001
Female			
CTI as continuous	1.56 (1.18-2.06), 0.002	1.52 (1.15-2.01), 0.003	1.48 (1.12-1.96), 0.006
CTI quartile			
Q1	1.00	1.00	1.00
Q2	1.24 (0.89-1.73), 0.204	1.21 (0.87-1.69), 0.258	1.18 (0.84-1.65), 0.341
Q3	1.76 (1.28-2.42), 0.001	1.65 (1.20-2.27), 0.002	1.54 (1.12-2.12), 0.008
Q4	2.68 (1.97-3.65), <0.0001	2.38 (1.75-3.24), <0.0001	2.21 (1.60-2.99), <0.0001
P for trend	<0.0001	<0.0001	<0.0001

Model 1: unadjusted.

Model 2: adjusted for age and sex.

Model 3: adjusted for the same variables as model 2, with additional covariates, including LDL, diabetes, SBP, HDL, NEUT, FIB-4, WBC, LYM, DBP, AST, ALT and hypertension. The P for interaction between CTI and sex was 0.02, calculated by adding a multiplicative CTI × sex term to the fully adjusted model.

Sex-stratified analyses demonstrated independent associations in both men (fully adjusted OR 1.82, 95% CI 1.40–2.36) and women (OR 1.48, 95% CI 1.12–1.96), with a stronger effect magnitude in men. Formal interaction testing was performed by adding a CTI × sex term to the fully adjusted model. The CTI × sex interaction was statistically significant (P for interaction = 0.02), supporting sex-related effect modification in the association between CTI and obstructive CAD.

### 3.4 Discriminative performance of CTI in overall and sex-stratified analyses

**Figure 2:**
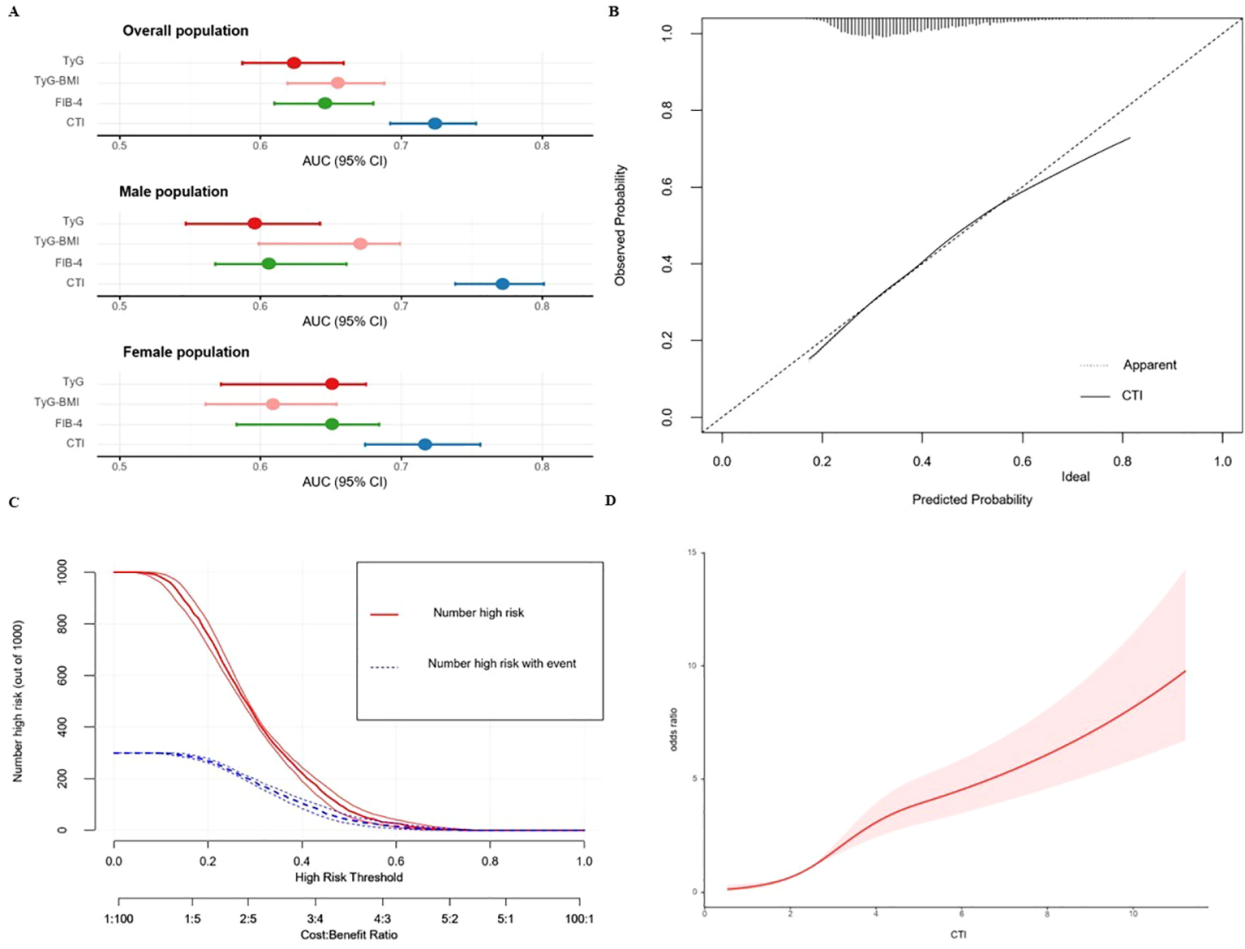
Discriminative performance, calibration, clinical impact, and dose–response relationship of CTI for obstructive coronary artery disease in patients with MASLD. **(A)** Comparison of the area under the receiver operating characteristic curve (AUC) with 95% confidence intervals for CTI, TyG, TyG-BMI, and FIB-4 in the overall population and stratified by sex. **(B)** Calibration curve of the CTI-based logistic regression model for obstructive coronary artery disease. The dashed diagonal line represents perfect agreement between predicted and observed probabilities, whereas the solid line indicates the apparent calibration of the model. **(C)** Clinical impact curve (CIC) of the CTI-based model, illustrating the estimated number of individuals classified as high risk (red solid line) and the number of true-positive cases among those classified as high risk (blue dashed line) across different risk thresholds. **(D)** Restricted cubic spline (RCS) analysis showing the dose–response association between CTI and the odds of obstructive coronary artery disease. The red solid line represents the adjusted odds ratio, and the shaded area indicates the 95% confidence interval.

Discrimination analyses showed that CTI had higher apparent discriminative performance than FIB-4, TyG-BMI, and TyG for obstructive CAD. The AUC of CTI was 0.724 (95% CI 0.692–0.753) in the overall population, 0.772 (95% CI 0.738–0.801) in men, and 0.717 (95% CI 0.674–0.756) in women (**Supplementary Table 2**; **Figure 2A**). Calibration analysis showed acceptable agreement between predicted and observed probabilities for the CTI-based logistic model (**Figure 2B**). Clinical impact analysis illustrated the estimated number of individuals classified as high risk and the number of high-risk individuals with obstructive CAD across threshold probabilities (**Figure 2C**). RCS analysis further supported a graded positive association between CTI and the odds of obstructive CAD (**Figure 2D**) with a steeper risk gradient in men (**Supplementary Figure 3**). Reclassification analyses showed that CTI provided modest incremental value compared with TyG, TyG-BMI, and FIB-4. This improvement was reflected by higher AUCs and generally positive NRI and IDI estimates, particularly in men. In women, the incremental metrics were weaker and less consistent, indicating that the sex-stratified reclassification results should be interpreted cautiously (**Table 3**).

**Table 3:** Comparative reclassification metrics of CTI and conventional indices for obstructive coronary artery disease in patients with MASLD.

Population	Comparison	Continuous NRI, index (95% CI)	P value	IDI, index (95% CI)	P value
Overall	CTI vs TyG	0.186 (0.092, 0.280)	<0.001	0.018 (0.007, 0.029)	0.002
Overall	CTI vs TyG-BMI	0.132 (0.041, 0.223)	0.004	0.012 (0.003, 0.022)	0.011
Overall	CTI vs FIB-4	0.154 (0.061, 0.247)	0.001	0.015 (0.005, 0.026)	0.004
Male	CTI vs TyG	0.238 (0.118, 0.358)	<0.001	0.024 (0.010, 0.038)	0.001
Male	CTI vs TyG-BMI	0.176 (0.062, 0.290)	0.003	0.017 (0.005, 0.030)	0.006
Male	CTI vs FIB-4	0.226 (0.107, 0.345)	<0.001	0.022 (0.009, 0.036)	0.001
Female	CTI vs TyG	0.096 (0.003, 0.189)	0.043	0.008 (0.001, 0.017)	0.039
Female	CTI vs TyG-BMI	0.124 (0.027, 0.221)	0.012	0.011 (0.002, 0.021)	0.018
Female	CTI vs FIB-4	0.089 (-0.006, 0.184)	0.066	0.007 (-0.001, 0.016)	0.081

### 3.5 Subgroup and sensitivity analyses

Subgroup analyses showed generally consistent associations across age, diabetes, and hypertension strata (all P for interaction >0.05; **Supplementary Figure 4**). The positive association persisted in both diabetic and non-diabetic subgroups, with comparable effect sizes observed among normotensive participants. Sensitivity analyses showed broadly consistent findings. When obstructive CAD was alternatively defined as ≥70% luminal stenosis, CTI remained positively associated with CAD in the overall population and in sex-stratified analyses. The association was generally stronger in men than in women, consistent with the primary analysis (**Supplementary Table 4**).

### 3.6 Prognostic value of CTI in NHANES MASLD population

In the NHANES MASLD cohort, baseline characteristics differed significantly according to survival status (**Supplementary Table 5**). Non-survivors were older, had a higher prevalence of diabetes and hypertension, and exhibited higher CTI levels than survivors, suggesting a potential association between elevated CTI and adverse long-term outcomes.

In Cox proportional hazards analyses (**Supplementary Table 6**), higher CTI was consistently associated with increased risks of both all-cause and CVD mortality in individuals with MASLD. These associations remained significant after sequential adjustment for demographic factors, lifestyle variables, and cardiometabolic risk factors. Sex-stratified analyses demonstrated that CTI was independently associated with all-cause mortality in both men and women. For CVD mortality, the association remained robust in men across fully adjusted models, whereas in women the association was attenuated and no longer statistically significant after full adjustment.

## 4 Discussion

In this study, CTI was associated with CCTA-defined obstructive CAD after multivariable adjustment. Machine-learning-assisted feature selection and stability analyses consistently prioritized CTI among candidate clinical variables, supporting its analytical stability rather than establishing a standalone predictive model. CTI also showed moderate apparent discriminative performance and performed better than several comparator indices. The association appeared more pronounced in men, and formal interaction testing supported potential sex-related effect modification. In the NHANES cohort, CTI was associated with long-term all-cause and cardiovascular mortality, providing external prognostic context rather than external validation for CCTA-defined CAD.

The observed association between CTI and obstructive CAD likely reflects its unique capacity to integrate two core drivers of atherosclerosis in MASLD: insulin resistance and chronic low-grade inflammation. While the TyG index captures metabolic stress and CRP reflects systemic inflammation, their combination into a single metric may synergistically capture the reciprocal amplification between these pathways (29, 30). Insulin resistance exacerbates endothelial dysfunction and promotes a pro-inflammatory state, while inflammation further impairs insulin signaling, creating a vicious cycle that accelerates plaque formation. As a composite index, CTI may partially reflect the systemic burden of disease than either component alone, providing a plausible explanation for its superior discriminative performance and its association with long-term mortality. The relatively modest performance of FIB-4 in our cohort likely reflects the early disease stage of our population, where fibrotic burden is low and metabolic-inflammatory drivers predominate. CTI may therefore be better suited for early cardiovascular risk stratification in MASLD, while FIB-4 retains utility in advanced liver disease.

An intriguing finding of our study is the sex-specific difference in CTI’s performance, with stronger discriminative ability observed in men. This contrasts with some previous studies reporting enhanced associations of TyG-related indices with cardiovascular risk in women (31). Several factors may account for this discrepancy. First, the incorporation of CRP may amplify sex-specific inflammatory pathways; men typically exhibit higher baseline CRP levels and greater inflammatory responsiveness to metabolic stress, potentially strengthening the CTI-CAD association in males. Second, hormonal differences play a critical role—estrogen confers vascular protection in premenopausal women, which may attenuate the impact of insulin resistance-driven atherosclerosis (32, 33). Third, the higher burden of traditional cardiovascular risk factors in our male cohort may have enhanced the correlation between CTI and CAD outcomes. Collectively, these findings suggest that while insulin resistance indices alone may perform differently across sexes, the integration of inflammation (as in CTI) may shift the discriminative sensitivity toward a phenotype more prevalent in men. These findings support further investigation of sex-related differences in CTI-based cardiovascular risk stratification in MASLD. To our knowledge, this study is among the first to evaluate the association between CTI, an integrated metabolic–inflammatory index, and CCTA-defined obstructive CAD in patients with MASLD. Using multivariable regression, discrimination analyses, and machine-learning-assisted feature selection, we found that CTI was consistently associated with obstructive CAD and showed better discriminative performance than several conventional indices. The machine-learning-assisted analyses were intended to assess feature stability rather than to develop a standalone prediction model. Sex-stratified analyses suggested stronger associations and discriminative performance in men, but these findings should be interpreted cautiously and require confirmation in independent cohorts. The NHANES analysis further suggested that CTI was associated with long-term mortality in a population-based MASLD cohort, providing prognostic context rather than external validation of CCTA-defined CAD. Several limitations should also be acknowledged. First, the cross-sectional design precludes causal inference, and the discriminative implications of CTI for incident cardiovascular events warrant prospective validation. Second, although our analyses were adjusted for major confounders, residual or unmeasured factors—such as medication use, sex hormones, or lifestyle behaviors—cannot be fully excluded. Third, external prognostic validation in independent cohorts and inclusion of longitudinal outcome data would further strengthen the clinical applicability of CTI. Moreover, while the use of CRP, a widely available and low-cost marker, enhances the clinical translatability of our findings, it is a non-specific inflammatory marker. Future studies incorporating more specific inflammatory signatures (e.g., IL-6, TNF-α) or adipokines may better delineate the mechanistic basis of the observed CTI-CAD association. The imaging-based cohort was derived from a single tertiary center and included MASLD patients referred for CCTA because of suspected CAD. Therefore, our findings apply primarily to a high-risk clinical population and may not be directly generalizable to asymptomatic or community-based MASLD populations. Although CCTA provides an objective anatomical assessment of coronary stenosis, future multicenter prospective studies including broader MASLD populations are warranted to validate the clinical applicability of CTI.

## 5 Conclusion

In summary, CTI was independently associated with obstructive CAD in patients with MASLD and showed moderate apparent discriminative performance compared with traditional metabolic markers, particularly in men. Moreover, elevated CTI was associated with increased risks of all-cause and cardiovascular mortality, supporting its potential value for cross-sectional risk stratification and long-term prognostic assessment in MASLD.

## Data Availability

The raw data supporting the conclusions of this article will be made available by the authors, without undue reservation.
