# Supplementary material for: Sex differences in the association of the CRP-TyG index with coronary artery disease and long-term mortality in MASLD
Source: Front Endocrinol (Lausanne). 2026 Jul 28;17:1864536. doi: 10.3389/fendo.2026.1864536 (PMC13457702; doi:10.3389/fendo.2026.1864536)
Supplement: Supplementary file 1 [file SupplementaryFile1.docx]

Supplementary material

Supplementary method:

1.CT scanning protocols

A bolus of 60–80 mL of iohexol contrast agent was administered via an antecubital vein at a flow rate of 5 mL/s, followed by a 50 mL saline flush at the same rate. Scan initiation was determined by bolus tracking, with the region of interest placed at the level of the ascending aorta and a trigger threshold set between 90 and 100 Hounsfield units (HU). After a 6-second delay, image acquisition was performed over a period of 5–12 seconds. The scan range extended from 1 cm below the carina to 1.5 cm below the inferior cardiac border. The imaging parameters included a tube current of 280–350 mA and a tube voltage of 120 kV.

2. Diagnosis of MASLD

The diagnosis of Metabolic Dysfunction-Associated Steatotic Liver Disease (MASLD) was established in accordance with international consensus criteria, requiring the co-presence of hepatic steatosis, detected by imaging or histology, and at least one of the following cardiometabolic risk factors: body mass index ≥25 kg/m², fasting plasma glucose ≥100 mg/dL (5.6 mmol/L) or HbA1c ≥5.7% or antidiabetic treatment, blood pressure ≥130/85 mmHg or antihypertensive medication, fasting triglycerides ≥150 mg/dL (1.7 mmol/L) or lipid-lowering treatment, or high-density lipoprotein cholesterol <40 mg/dL (1.0 mmol/L) for men and <50 mg/dL (1.3 mmol/L) for women or lipid-lowering treatment, all in the absence of significant alcohol consumption (<30 g/day for men, <20 g/day for women) and other competing chronic liver diseases.

3. Feature selection and stability analysis

Feature selection analyses were conducted using data from the imaging-based MASLD cohort. To identify the most informative features of CAD in patients with MASLD, a multi-method feature selection strategy was implemented to enhance robustness and minimize model dependency. All analyses were performed in R using complementary algorithms—least absolute shrinkage and selection operator (LASSO), Boruta, extreme gradient boosting (XGBoost), and support vector machine–recursive feature elimination (SVM-RFE)—to ensure that both linear and nonlinear relationships were adequately captured.

LASSO regression was applied using the glmnet package with tenfold cross-validation to determine the optimal penalty parameter. Variables with nonzero coefficients at the minimum lambda were considered informative and ranked according to the absolute magnitude of their coefficients. The Boruta algorithm, based on a random forest wrapper, iteratively compared the importance of each variable with randomly permuted shadow features, confirming those that consistently outperformed their shadows after 500 iterations. In parallel, an XGBoost model was trained using gradient boosting of decision trees with a binary logistic objective. Variable importance was quantified by the average information gain and used to derive ranked lists of features. Additionally, SVM-RFE was employed as a wrapper-based method to iteratively eliminate the least important features based on the weight magnitude derived from a linear support vector machine. This approach provided a complementary perspective on feature relevance, particularly in capturing margin-based discriminative patterns.

To evaluate the stability and reproducibility of feature selection, a subsampling-based framework was implemented. In each iteration, 70% of the original dataset was randomly subsampled without replacement to generate a training subset, within which the four algorithms (LASSO, Boruta, XGBoost, and SVM-RFE) were independently executed. This procedure was repeated 1,000 times to compute the selection frequency of each variable across resamples.

The selection frequencies from the four algorithms were aggregated to derive an overall stability index for each feature, reflecting its robustness across different analytic strategies. Bar plots displaying the aggregated selection frequencies of the top 20 variables and cumulative stability curves illustrating the mean inclusion frequency of the CTI index were generated to visualize its reproducibility relative to random selection.

This multi-algorithm, subsampling-based framework was designed to enhance the reliability of feature identification and to ensure that stable feature such as the CTI index were recognized based on reproducibility rather than algorithm-specific bias.

Supplementary Table 1. Baseline Characteristics of the Study Population, Stratified by Sex.

| Variables | Total (n = 2119) | Female (n = 913) | Male (n = 1206) | P value |
| --- | --- | --- | --- | --- |
| Age, years | 52.69 ± 14.41 | 56.91 ± 12.98 | 49.49 ± 14.62 | <0.001 |
| BMI | 25.57 ± 1.81 | 24.84 ± 1.89 | 26.31 ± 1.58 | 0.01 |
| SBP, mmHg | 132.37±0.51 | 130.29±0.73 | 134.83±0.77 | < 0.0001 |
| DBP, mmHg | 67.81±0.37 | 66.06±0.50 | 69.29±0.50 | < 0.0001 |
| Smoking, n(%) | 784 (37.0) | 142 (15.6) | 642 (53.2) | <0.001 |
| Medical history |  |  |  |  |
| Hypertension, n(%) | 1443 (68.1) | 482 (52.8) | 961 (79.7) | <0.001 |
| Diabetes, n(%) | 835 (39.4) | 314 (34.4) | 521 (43.2) | <0.001 |
| Hyperlipidemia, n(%) | 473 (22.3) | 134 (14.7) | 339 (28.1) | <0.001 |
| Laboratory test |  |  |  |  |
| WBC(× 109/L) | 7.55±0.05 | 7.56±0.08 | 7.55±0.06 | 0.88 |
| PLT(× 109/L) | 228.01±1.63 | 245.15±2.43 | 213.14±1.98 | < 0.0001 |
| NEUT(× 109/L) | 4.59±0.04 | 4.58±0.06 | 4.59±0.05 | 0.88 |
| LYM(× 109/L) | 2.05±0.03 | 2.12±0.05 | 2.00±0.04 | 0.11 |
| FPG, mmol/L | 6.80 ± 3.09 | 6.70 ± 2.82 | 6.88 ± 3.28 | 0.036 |
| HbA1c, % | 7.48 ± 1.89 | 7.42 ± 1.76 | 7.52 ± 1.97 | 0.283 |
| TC, mmol/L | 4.86 ± 1.26 | 4.94 ± 1.17 | 4.81 ± 1.32 | <0.001 |
| TG, mmol/L | 1.85 (1.27, 2.80) | 1.71 (1.23, 2.50) | 1.96 (1.32, 3.05) | <0.001 |
| LDL, mmol/L | 2.91 ± 0.87 | 2.97 ± 0.85 | 2.87 ± 0.89 | <0.001 |
| HDL, mmol/L | 1.04 ± 0.30 | 1.11 ± 0.28 | 0.99 ± 0.30 | <0.001 |
| ALT, U/L | 31.00 (20.80, 48.00) | 25.20 (18.00, 38.00) | 35.90 (23.20, 56.00) | <0.001 |
| AST, U/L | 23.30 (18.00, 32.77) | 22.00 (17.80, 30.00) | 24.40 (19.00, 34.00) | <0.001 |
| ALB, g/L | 42.68 ± 4.64 | 42.13 ± 4.43 | 43.09 ± 4.75 | <0.001 |
| TBIL, umol/L | 13.19 ± 14.42 | 11.63 ± 11.91 | 14.36 ± 15.96 | <0.001 |
| GGT, U/L | 37.95 (25.00, 61.95) | 29.00 (21.00, 43.00) | 47.00 (31.00, 75.00) | <0.001 |
| TBA, umol/L | 3.50 (2.00, 5.80) | 3.30 (1.90, 5.50) | 3.70 (2.10, 6.00) | <0.001 |
| UA, umol/L | 345.00 (280.00, 414.00) | 309.00 (256.00, 365.00) | 378.00 (310.00, 447.00) | <0.001 |
| Creatinine, umol/L | 64.00 ± 35.56 | 53.15 ± 20.36 | 72.22 ± 41.86 | <0.001 |
| CRP, mg/L | 6.22±0.03 | 6.18±0.04 | 6.55±0.08 | 0.01 |
| TyG | 8.86±0.03 | 8.84±0.03 | 9.18±0.04 | <0.001 |
| TyG-BMI | 226.55 ± 16.3 | 219.59 ± 16.8 | 241.53 ± 14.1 | <0.001 |
| FIB-4 | 1.34 ± 0.82 | 1.17 ± 0.45 | 1.47 ± 1.02 | <0.001 |
| CTI | 9.20 ± 0.76 | 9.10 ± 0.69 | 9.27 ± 0.81 | <0.001 |
| CAD, n(%) | 495 (23.3) | 169 (18.5) | 326 (27) | <0.001 |

Supplementary Table 2 Area under the curve of CTI for Obstructive Coronary Artery Disease in the whole population and in both women and men groups

|  | AUC | 95%CI |
| --- | --- | --- |
| Overall |  |  |
| CTI | 0.724 | 0.692-0.753 |
| FIB-4 | 0.646 | 0.610-0.680 |
| TyG-BMI | 0.655 | 0.619-0.688 |
| TyG | 0.624 | 0.587-0.659 |
| Male |  |  |
| CTI | 0.772 | 0.738-0.801 |
| FIB-4 | 0.606 | 0.568-0.661 |
| TyG-BMI | 0.671 | 0.599-0.699 |
| TyG | 0.596 | 0.547-0.642 |
| Female |  |  |
| CTI | 0.717 | 0.674-0.756 |
| FIB-4 | 0.651 | 0.583-0.684 |
| TyG-BMI | 0.609 | 0.561-0.654 |
| TyG | 0.651 | 0.572-0.675 |

Supplementary Table 3. Subgroup analysis

|  | OR, 95%CI | P value |
| --- | --- | --- |
| Age≥60 |  |  |
| Male | 3.35 (2.40 - 4.68) | <0.0001 |
| Female | 2.30 (1.65 - 3.21) | <0.0001 |
| **Age < 60 years** |  |  |
| Male | 2.95 (2.08 - 4.18) | <0.0001 |
| Female | 2.05 (1.48 - 2.84) | <0.0001 |
| **With Diabetes** |  |  |
| Male | 3.52 (2.45 - 5.06) | <0.0001 |
| Female | 2.45 (1.72 - 3.49) | <0.0001 |
| **Without Diabetes** |  |  |
| Male | 3.02 (2.20 - 4.15) | <0.0001 |
| Female | 2.10 (1.55 - 2.85) | <0.0001 |
| **With Hypertension** |  |  |
| Male | 3.41 (2.50 - 4.65) | <0.0001 |
| Female | 2.35 (1.73 - 3.19) | <0.0001 |
| **Without Hypertension** |  |  |
| Male | 2.88 (2.02 - 4.11) | <0.0001 |
| Female | 1.98 (1.42 - 2.76) | <0.0001 |

Supplementary Table 4. Sensitivity analyses using an alternative definition of obstructive CAD

| Population | CAD Definition | OR | 95%CI | P value |
| --- | --- | --- | --- | --- |
| Overall | ≥50% stenosis | 1.68 | 1.33–2.12 | <0.001 |
| Overall | ≥70% stenosis | 1.51 | 1.31–2.65 | <0.001 |
| Male | ≥50% stenosis | 1.82 | 1.40–2.36 | <0.001 |
| Male | ≥70% stenosis | 1.72 | 1.41-2.81 | <0.001 |
| Female | ≥50% stenosis | 1.48 | 1.12–1.96 | <0.001 |
| Female | ≥70% stenosis | 1.37 | 1.19-2.11 | 0.002 |

Supplementary table 5 Baseline characteristics according to survival status in NHANES cohort

| Variable | Total | Survivors | Non-survivors | P-value |
| --- | --- | --- | --- | --- |
| Age, years | 53.27(0.62) | 50.41(0.63) | 68.38(1.28) | < 0.0001 |
| Sex, % |  |  |  | 0.4 |
| Female | 42.38 | 43 | 39.06 |  |
| Male | 57.62 | 57 | 60.94 |  |
| Ethnicity |  |  |  | 0.01 |
| Mexican American | 11.79 | 12.99 | 5.46 |  |
| Non-Hispanic Black | 5.45 | 5.57 | 4.81 |  |
| Non-Hispanic White | 72.41 | 70.31 | 83.52 |  |
| Other Hispanic | 5.4 | 5.75 | 3.53 |  |
| Other Race | 4.95 | 5.38 | 2.69 |  |
| Smoke, % |  |  |  | < 0.001 |
| Former | 31.29 | 29.35 | 45.48 |  |
| Never | 53.96 | 57.81 | 40.86 |  |
| Now | 12.7 | 12.83 | 13.66 |  |
| DM, % | 31.87 | 27.36 | 56.45 | < 0.001 |
| Hypertension, % | 54.89 | 51.36 | 73.55 | < 0.001 |
| BMI, kg/m^2^ | 33.47(0.23) | 33.73(0.25) | 32.07(0.54) | 0.01 |
| Glucose, mg/dl | 119.18(1.17) | 115.90(0.82) | 136.50(5.36) | < 0.001 |
| HbA1c | 5.96(0.04) | 5.90(0.03) | 6.30(0.13) | 0.004 |
| Triglyceride, mmol/L | 1.90(0.05) | 1.92(0.05) | 1.84(0.12) | 0.53 |
| Total cholesterol, mmol/L | 5.06(0.05) | 5.11(0.06) | 4.81(0.07) | 0.001 |
| HDL, mmol/L | 1.18(0.01) | 1.17(0.01) | 1.24(0.03) | 0.04 |
| LDL, mmol/L | 3.03(0.04) | 3.09(0.05) | 2.76(0.07) | < 0.001 |
| Albumin, g/L | 41.88(0.10) | 42.03(0.12) | 41.07(0.19) | < 0.001 |
| ALT, U/L | 30.69(0.75) | 31.79(0.76) | 24.83(1.27) | < 0.0001 |
| AST, U/L | 27.43(0.63) | 27.63(0.68) | 26.41(1.00) | 0.27 |
| WBC(× 10^9^/L) | 7.16(0.05) | 7.10(0.06) | 7.51(0.22) | 0.09 |
| LYM(× 10^9^/L) | 2.08(0.02) | 2.10(0.03) | 1.92(0.05) | 0.01 |
| Hb(× 10^9^/L) | 14.58(0.07) | 14.65(0.08) | 14.19(0.14) | 0.01 |
| PLT(× 10^9^/L) | 256.10(2.31) | 258.07(3.18) | 245.79(7.84) | 0.22 |
| NEU(× 10^9^/L) | 4.28(0.05) | 4.20(0.06) | 4.71(0.18) | 0.02 |
| Creatinine, umol/L | 79.56(0.88) | 77.51(0.86) | 90.41(2.65) | < 0.0001 |
| Uric acid, umol/L | 364.53(2.98) | 360.54 (3.30) | 385.56(10.37) | 0.03 |
| CTI | 10.14(0.05) | 10.12(0.05) | 11.35 (0.20) | <0.001 |

Categorical variables were presented as weighted proportions.

Supplementary table6 The associations of CTI with all-cause and CVD mortality in individuals with MASLD

|  | HRs (95%CI), P-value | | |
| --- | --- | --- | --- |
|  | Model 1, p-value | Model 2, p-value | Model 3, p-value |
| All-cause mortality |  |  |  |
| Overall | 1.21(1.07,1.51), <0.001 | 1.08(1.02,1.32), 0.002 | 1.06(1.01,1.26), 0.01 |
| Male | 1.28(1.08,1.69), <0.001 | 1.15(1.02,1.44), 0.001 | 1.12(1.08,1.50), 0.02 |
| Female | 1.18(1.05,1.79), <0.001 | 1.09(1.01,1.52),0.002 | 1.04(1.01,1.36), 0.01 |
| CVD mortality |  |  |  |
| Overall | 1.17(1.05, 1.63), <0.001 | 1.15(1.09,1.48), 0.003 | 1.05(1.02,1.38), 0.04 |
| Male | 1.19(1.02,1.78), <0.001 | 1.17(1.04,1.66), 0.001 | 1.11(1.03,1.31), 0.03 |
| Female | 1.11(1.03,1.69), <0.001 | 1.09(1.02,1.61), 0.003 | 1.06(0.91,1.43), 0.11 |

Note: Cox regression analysis: Model 1: No adjusted. Model 2: Adjusted for covariates including age, ethnicity. Model 3: adjusted for the same variables as model 2, with additional covariates, including LDL, diabetes, SBP, HDL, NEUT, FIB-4, WBC, LYM, TC, DBP, and hypertension.

Supplementary Fig. 1. Overall study design, participant selection, and analytical workflow.

Flow diagram summarizing participant inclusion and exclusion, feature selection, model construction, and sensitivity analyses in the MASLD cohort.

Supplementary Fig. 2. Bootstrap-based feature selection frequency analysis.

(A) Cumulative inclusion frequency curve of CTI across 1,000 bootstrap resamples in the overall population. (B–C) Top 20 features ranked by selection frequency in male (B) and female (C) subgroups, respectively. (D–E) Cumulative inclusion frequency curves of CTI across 1,000 bootstrap resamples in male (D) and female (E) populations.

Supplementary Figure 3. Sex-stratified RCS curves illustrating the relationship between CTI and the OR of obstructive CAD in male and female participants.

Supplementary Fig. 4 Stratified analyses assessing the association between CTI and CAD across subgroups defined by (A) age, (B) DM, and (C) hypertension.
